# Mixed Polymer Brushes for “Smart” Surfaces

**DOI:** 10.3390/polym12071553

**Published:** 2020-07-13

**Authors:** Mingxiao Li, Christian W. Pester

**Affiliations:** 1 Department of Chemical Engineering, The Pennsylvania State University, University Park, PA 16802, USA; mul444@psu.edu; 2 Department of Materials Science and Engineering, The Pennsylvania State University, University Park, PA 16802, USA; 3 Department of Chemistry, The Pennsylvania State University, University Park, PA 16802, USA

**Keywords:** polymer brushes, responsive, materials, surfaces, surface coating, surface-initiated polymerization, controlled radical polymerization, phase segregation, external regulation

## Abstract

Mixed polymer brushes (MPBs) are composed of two or more disparate polymers covalently tethered to a substrate. The resulting phase segregated morphologies have been extensively studied as responsive “smart” materials, as they can be reversible tuned and switched by external stimuli. Both computational and experimental work has attempted to establish an understanding of the resulting nanostructures that vary as a function of many factors. This contribution highlights state-of-the-art MPBs studies, covering synthetic approaches, phase behavior, responsiveness to external stimuli as well as novel applications of MPBs. Current limitations are recognized and possible directions for future studies are identified.

## 1. Introduction

Polymer surface coatings find various applications ranging from household products to nanotechnology devices. Although physisorbed polymer layers (e.g., spray and dip coating) are versatile in their chemistry, there exist specific limitations: physisorbed coatings inherently lack the ability to reversibly and intelligently respond to their environment (e.g., solvents) and can be susceptible to detachment from the surface upon exposure to solvents (de-wetting). Here, covalently surface-tethered macromolecules, or polymer brushes, offer a unique alternative platform for coating surfaces [1,2]. The chemical connection between polymer and substrate improves durability and provides the potential for stimuli-responsive properties [3]. For detailed reviews on homopolymer brushes, the reader is referred to a series of previous articles [1,2,4,5,6,7,8,9,10,11,12,13,14]. Using different anchoring chemistries, polymer brushes can coat various surfaces, including silicon [15], silica [16], gold [17], metal oxides [18], polymers [19], etc., and various surface geometries including planar substrates [20], nano particles [10] and free-standing films [21]. Polymer brush films therefore provide the potential to not only improve household goods but also address larger-scale issues such as anti-fouling and drug delivery [22,23]. Potential applications span a wide range [2,24,25,26,27,28,29] from organic light emitting diodes (OLEDs) [30] to membranes for desalination and gas separation [31], to protein adsorption [32]. Additional applications include those that require controlled surface wettability [33] and heterogeneous catalysis [34]. The tethering of one of the polymer’s chain ends introduces the ability to respond to external stimuli [3], e.g., pH [35,36], ion strength [35,36], oxidoreduction [37], solvent [38], temperature [39], light [40], electric [41], and magnetic fields [42].

**Figure 1:**
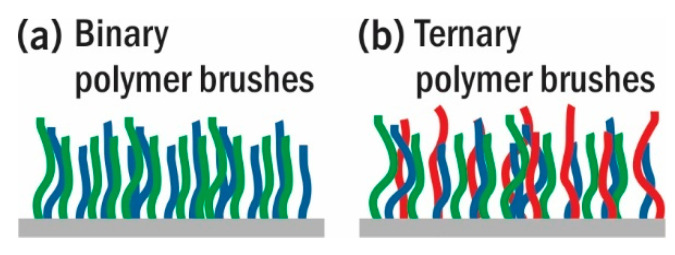
Schematic illustration of binary and ternary MPBs.

Mixed polymer brushes (MPBs) are polymer brushes composed of two (binary MPBs), three (ternary MPBs [43,44]), or more disparate polymers which are randomly co-tethered to the surface (see Figure 1). MPBs have drawn interest because of their unique ability to selectively respond to their external environment. In contrast to deposition of multiple polymers from solution or melt (polymer blends), the covalent bond allows for phase segregation on the nanoscale and prevents de-wetting. As a result, complex phase diagrams have been proposed and theoretically described. Much work suggests an ability to respond to external stimuli as a result of polymer chains minimizing their free energy, leading to the ability to selectively target or modify surface compositions and physical properties of the engineered substrates. As such, MPB can be considered as a potent way to manufacture “smart” surfaces with switchable physical properties for varied applications including, but not limited to, drug release [45], catalysis [46], nanomotors [47], nanovalves [48], and adsorption/desorption of polymers [49] or proteins [50,51,52,53,54].

With increased interest in dynamic and responsive surfaces, this review surveys previous work, recognizes current challenges, and provides an outlook for future directions in the field of MPBs. It is important to note that we primarily focus on planar substrates; MPBs on curved surfaces and particles have been reviewed previously and provide promising platforms for materials that can be selectively shuttled between phases in multi-component mixtures [55]. Note that there are also so-called “binary” or “ternary” polymer brushes, patterned manually in nanometer- or micron-scales (e.g., using a photomask) on a surface [56,57,58,59,60,61,62,63,64]. We do not consider these as “binary or ternary *mixed* polymer brushes” here, since their tethered ends are not inherently mixed on a molecular level. After a description of synthetic routes, we will discuss the phase behavior of binary and ternary MPBs before we describe the switching of topographies using external stimuli. We end this review by elaborating on previously established and suggested applications as an outlook for future work.

## 2. Synthetic Routes

**Figure 2:**
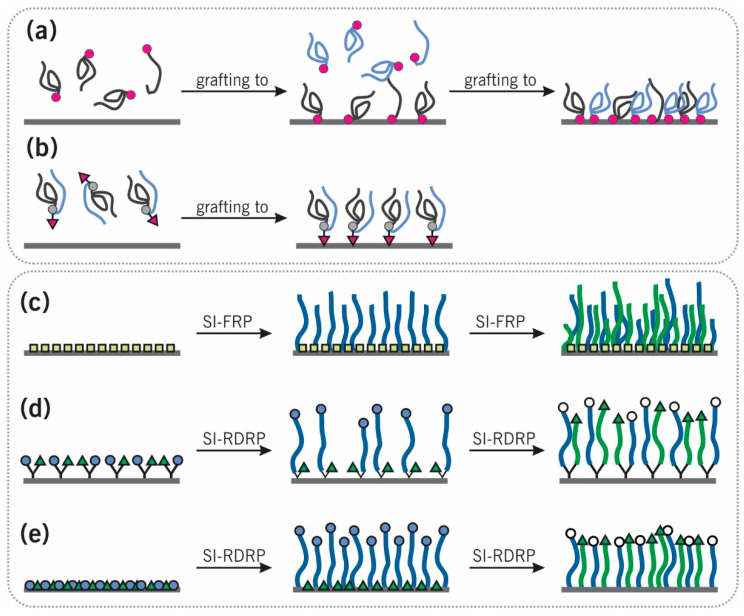
Schematic illustration of different synthetic routes. (**a**) Step-wise “grafting to” of individual homopolymers, (**b**) “grafting to” of Y-shaped diblock copolymers, (**c**) step-wise “grafting from” via surface-initiated free-radical polymerization (SI-FRP) using non-selective initiators, (**d**) “grafting from” via surface-initiated reversible-deactivation radical polymerization (SI-RDRP) using two disparate co-deposited initiators, and (**e**) “grafting from” via SI-RDRP using Y-shaped bifunctional initiators.

There exist two recognized methods for manufacturing homopolymer and mixed polymer brushes: “grafting to” and “grafting from” (see Figure 2). The former refers to attaching previously synthesized polymer chains to a surface, while the latter involves initiating the growth of polymer brushes directly from a surface that was previously functionalized with initiator monolayers [65]. Each approach comes with its own set of advantages and disadvantages: while “grafting to” benefits from facile and precise polymer characterization prior to surface deposition, obtainable grafting densities are limited by the polymer’s radius of gyration (*R*_g_) and its diffusion to the grafting site. In contrast, “grafting from” (i.e., surface-initiated polymerization) requires diffusion of only small monomers to an actively growing chain end on the surface. As a result, greater polymer grafting densities can be achieved via “grafting from” than via “grafting to” due to the improved mass transport and decreased steric hindrance [1,2,11,13,14,66,67].

### 2.1. “Grafting to”

“Grafting to” can be accomplished following two methods: (i) sequential or simultaneous co-deposition of two individual types of chain (see Figure 2a) or (ii) deposition of a “Y-shaped” diblock copolymer with a functional group located among the two disparate polymer segments (see Figure 2b). The Y-shaped diblock copolymer method inherently fixes the ratio of surface-tethered polymers: each surface-grafting site is automatically functionalized with both polymers and ensures that the two polymers in close proximity at a molecular level. In contrast, the co-deposition of individual chains provides additional flexibility to tune the grafting ratio of the two disparate components [50,68]. For the sequential “grafting to” approach (see Figure 2a), the order of functionalization can be critical. As an example, Minko et al. [69] reported synthesis of a polystyrene (PS)/poly(2-vinylpyridine) (P2VP) MPB using a *sequential* “grafting to” approach. Silicon wafers were functionalized with 3-glycidoxypropyltrimethoxysilane (GPS) whose epoxy functionality was subsequently reacted with carboxy-terminated PS through annealing at 150 °C. After rinsing off the untethered polymers, this experimental procedure was repeated for carboxy-terminated P2VP. As the two carboxy-terminated polymers compete for the same grafting sites, the ultimate fraction of the two components can be tuned by varying the annealing time of the first step. Intriguingly, upon investigating the inverse grafting order (P2VP first, followed by PS), only a few grafted PS chains were observed. In this study, sequential grafting was only effective if the polar polymer was grafted *after* the nonpolar component: the strong affinity between the polar 2-vinylpyridine monomer and the polar GPS layer allows the second reaction to penetrate the initial polymer chains and approach the substrate with residual reactive sites. In subsequent studies, weakly charged binary poly(acrylic acid) (PAA)/P2VP binary brushes were also studied [70].

Experimentally simpler, the “grafting to” route can be leveraged by co-depositing two pre-synthesized polymers simultaneously. For example, instead of sequential depositing, poly(ethylene oxide) PEO/PAA brushes were prepared by merging a gold substrate into a mixture of thiol-terminated PEO and PAA [50]. PEO and PAA chains were grafted simultaneously and the fraction of two components was controlled by the mass ratio in the mixture. It is important to note that, beyond the epoxy-carboxylic acid reaction and thiol–gold interaction, various reactions are available to create this chemical tether between polymer chains and the substrate [67,71,72,73,74]. 

By combining two different “anchoring” mechanisms, the selective “grafting to” of two polymers can be achieved. For example, two “click” reactions were applied to prepare poly(2-methacryloyloxyethyl phosphorylcholine) (PMPC)/poly(2-(methacryloyloxy) ethyl trimethylammonium chloride) (PMETA) mixed brushes [71]. A stainless-steel (SS) substrate was coated with a polydopamine (PDA) adhesive layer, which served as the initiator anchoring layer. Reactions of the resulting SS-PDA surface with ethylene sulfide and 3-azidopropyl carbonylimidazole introduced thiol and azide moieties on the PDA layer, respectively. Zwitterionic PMPC chains with terminal vinyl groups were subsequently grafted via thiol−ene “click” reaction, followed by cationic PMETA chains with alkyne groups via an azide−alkyne “click” reaction. The selectivity of these two grafting steps prevents the two chains from competing for the same grafting site.

Finally, binary MPBs can also be prepared by anchoring a functional group at the center of a diblock copolymer to a substrate (see Figure 2b) [75,76,77]. For example, Y-shaped PS-*b*-poly(*tert*-butyl acylate)(P*t*BA) diblock copolymers were synthesized from a trifunctional center [75,76]. Carboxy-terminated PS and P*t*BA were attached to two hydroxyl groups of the trifunctional center in a stepwise manner using a protection–coupling–deprotection strategy. The third functional group, a benzoic acid group, kept the capability of anchoring the Y-shaped diblock copolymer onto silicon wafers. MPBs prepared via Y-shape diblock copolymers strictly controls the grafting ratio of two polymers into 1:1. The two polymer chains are chemically connected on each grafting site. The Y-shaped MPBs minimize the grafting randomness which may disturb the long-range order of nanophase segregation [78,79]. 

### 2.2. “Grafting from”

“Grafting from” approaches, also called surface-initiated polymerizations (SI-P), have been extensively used to fabricate homopolymer brushes and mixed polymer brushes with high grafting densities. The reader is referred to excellent review articles surveying SI-P mechanisms [2,10]. Ever since seminal reports on SI-P [16,80], various researchers have worked to improve oxygen tolerance [60,81], user-friendliness [82], and introduce the ability to control polymer brush growth using external stimuli [81,83,84]. In the following, we will outline various SI-P combinations for MPB surface engineering. Figure 2c–e illustrate how the SI-P approach can be leveraged to synthesize MPBs.

#### 2.2.1. Sequential Surface-Initiated Free-Radical Polymerization (SI-FRP)

Sequential surface-initiated free-radical polymerization (SI-FRP [16]) has been widely used as a facile and fast approach to prepare MPBs – both binary [85] and ternary [43]. In general, an azo-initiator (e.g., 4,4′-azobis(4-cyanovaleric acid) (ACVA)) is tethered to a substrate to form a dense initiating layer. Subsequently, two SI-FRPs are performed from this functionalized substrate (see Figure 2c). Similar to the sequential “grafting to” of PS and P2VP (vide supra) [69], the sequence of SI-FRPs is important to consider: ellipsometry and Fourier transform infrared spectroscopy (FTIR) confirmed successful grafting of both components if SI-FRP of PS was performed first (followed by P2VP), however, inconclusive results were obtained for the reverse order (i.e., SI-FRP for P2VP first) followed by SI-FRP for PS [85]. The authors argue that this difference may be caused by the strong affinity of P2VP to the silica surface. This makes complete removal of physisorbed P2VP-“free” polymer formed in reaction solution challenging and prevents styrene monomer from approaching the residual initiating sites during subsequent SI-FRP of PS. Finally, ternary MPBs have also been fabricated via SI-FRPs by sequentially growing PS, poly(methyl methacrylate) (PMMA), and poly(4-vinyl pyridine) (P4VP) from ACVA-functionalized silicon wafers, sequentially (see Section 5) [43].

Although the sequential SI-FRP approach was used widely to fabricate MPBs (see Table 1), there are two major limitations for this approach: (i) Both polymerizations compete for the same surface initiator (e.g., ACVA) on the surface and (ii) limited control over polymer chain lengths and architectures due to the rapid uncontrolled propagation, high degree of side reactions, and termination during polymerization [86]. Mechanistically, the first SI-FRP may initiate most grafting sites and leave no active surface-initiators for the subsequent SI-FRP. Although the amount of residual initiators on the surface during the second step depends on reaction time of the first polymerization [85], a precise control on grafting ratios is still not achievable. These limitations have spawned research into sequential surface-initiated reversible-deactivation radical polymerizations (SI-RDRPs).

#### 2.2.2. Sequential Surface-Initiated Reversible-Deactivation Radical Polymerization (SI-RDRP)

In contrast to SI-FRP, SI-RDRP (often also referred to as controlled radical polymerization, CRP [115]), provides good control over polymer chain lengths and architectures through controlled chain propagation by reversible activation/deactivation of a functional chain end [116]. By carefully selecting two RDRP mechanisms, sequential polymerization can be performed without interfering with the respectively other type of initiator/active chain end [117,118,119,120]. This approach has been used successfully for both co-deposited (Figure 2e) and bifunctional Y-shaped (Figure 2d) RDRP initiator-functionalized surfaces. While the former provides flexibility in tuning grafting ratios of the two polymers, the latter ensures fixed 1:1 grafting ratios. The Y-shape initiators further avoid segregation of the two co-deposited initiators, guaranteeing that the two initiators are well-mixed on a molecular level within the monolayer. Especially on gold surfaces, the dynamic nature of the gold–thiol bond [13,121] can lead to organization and segregation of co-deposited initiators due to the, for example, reactivity, polarity, and steric hindrance.

The combination of surface-initiated atom transfer radical polymerization (SI-ATRP [122,123]) and surface-initiated nitroxide mediated radical polymerization (SI-NMP [124]) is one of most widely studied sequential SI-RDRP approaches. In seminal work, Zhao and coworkers [94,95] prepared PMMA/PS mixed brushes using sequential SI-ATRP/SI-NMP form both co-deposited [94] and Y-shaped surface-initiators [95]. SI-ATRP was performed to grow PMMA chains, followed by SI-NMP for PS chains. Free polymers formed from “sacrificial” initiators in solution were collected and characterized by gel permeation chromatography (GPC) to obtain polymer brush molecular weight estimates for both steps. While providing an estimate, it is worth noting that the molecular weight of free polymer does not necessarily reflect that of the actual tethered polymer brushes [125]. Successful fabrication of mixed PMMA/PS brushes was evidenced by ellipsometry and tensiometry. SI-ATRP was conducted first followed by SI-NMP, because the activation of an ATRP initiator with a metal complex is a bimolecular process, while the thermal activation of an NMP initiator follows a unimolecular pathway. This unimolecular activation is preferred as the second step because of the steric hindrance from the previously grown PMMA chains. Beyond PMMA/PS, Table 1 provides more examples for this sequential SI-ATRP/SI-NMP approach. Its popularity arises from the large monomer scope and versatility of ATRP, despite the second SI-NMP step being limited to styrenic monomers such as PS and P4VP.

Alternatively, SI-ATRP can be combined with surface-initiated reversible addition and fragmentation chain transfer polymerization (SI-RAFT [126]). Huang et al. [45] synthesized Y-shaped bifunctional surface-initiator composed of an ATRP initiator and a RAFT chain transfer agent (CTA) moiety on one grafting site. The Y-shaped initiators were tethered on aminopropyldimethylethoxysilane (APDES)-modified mesoporous silica nanoparticles (MSNs). Poly(*N*-(2-hydroxypropyl)methacrylamide) (PHPMA) was polymerized via SI-RAFT followed by extensive washing to remove physisorbed polymers. Then, the second polymer poly(2-diethylaminoethyl methacrylate) (PDEAEMA) was grafted via SI-ATRP. Again, both free RAFT CTA and sacrificial ATRP initiators were added to the reaction solution to obtain molecular weight estimates. 

It is important to note that, although SI-ATRP/SI-NMP can be performed successively without removing ATRP chain ends, it has been found that ATRP initiators may also extend during the SI-NMP step [95]. Thus, chain end deactivation (e.g., via chain end removal) is recommended for both sequential SI-ATRP/SI-NMP and SI-RAFT/SI-ATRP to avoid possible chain extension of the first polymer brush during the second polymerization [45,95]. For example, RAFT chain ends can be removed via a radical reaction with ACVA [45], and ATRP chain ends can be dehalogenated by tri-*n*-butyltin hydride (*n*-Bu_3_SnH) [95] or using light as an external stimulus [127,128]. To eliminate this additional synthetic step and further mitigate the possibility of undesired side reactions and cross-reactivity, research has moved towards orthogonal polymerization techniques. As successful orthogonal polymerizations of block copolymers and bottlebrush copolymers via RDRPs have been reported [117,118,119,120,129,130,131,132,133,134], we can foresee growing opportunities for fabricating MPBs via SI-RDRPs with improved orthogonality.

#### 2.2.3. Orthogonal SI-RDRP and Surface-Initiated Ring-Opening Polymerization (SI-ROP)

Synthetic approaches based on two orthogonal polymerizations provide a potent platform for MPB fabrication. For example, the combination of SI-RDRP and ring-opening polymerization (SI-ROP) has drawn extensive interest due to the specific monomer reactivity: while RDRP polymerizes vinyl monomers via a radical mechanism, ROP adds cyclic monomers following a ring-opening mechanism. Initiators for the two approaches cannot cross-interfere, respectively, with the other monomers. Furthermore, ROP inherently introduces backbone versatility to include as ester, ether, and amide functionalities. This orthogonal approach has found increasing adoption in solution polymerization [135,136,137] and can also provide possibilities to polymerize either chain end selectively as a function of external stimulation [138,139,140]. Recent research also shows how it is possible to reversibly switch between multiple mechanisms [141,142,143,144,145]. Nonetheless, for MPB synthesis, there exist only limited studies that leverage this orthogonal approach. Brittain and co-workers [114] reported a one-step one-pot synthesis of binary MPBs via a combination of SI-NMP and surface-initiated living cationic ring-opening polymerization (SI-CROP). A vinyl monomer, styrene, was polymerized via SI-NMP and a cyclic monomer 2-phenyl-2-oxazoline (PhOXA) was polymerized via SI-CROP, simultaneously. The successful growth of both polymers was observed by attenuated total reflection FTIR (ATR-FTIR). A surface switching behavior was confirmed after treatment with cyclohexane (selective solvent for PS) and methanol (selective solvent for PPhOXA) by X-ray photoelectron spectrometry (XPS) and tensiometry. Similarly, one-pot synthesis combining SI-ATRP and SI-ROP was reported to synthesize MPB on carbon nanotubes (CNTs) [112]. ROP and ATRP surface initiators were tethered onto multi-walled carbon nanotubes (MWNTs) via [4 + 2] Diels–Alder cycloaddition reactions. Then PS or PMMA was grown via SI-ATRP and poly(ε-caprolactone) (PεCL) or poly(l-lactic acid) (PLLA) was synthesized via SI-ROP.

## 3. Phase Behavior of Binary Mixed Brushes

### 3.1. Theoretical Discussion

As described in Flory and Huggins’ theory of polymer mixing, two immiscible homopolymers will undergo phase separation as a result of entropic and enthalpic considerations. If the two polymers are covalently connected, their (in)compatibility (as characterized by the Flory–Huggins interaction parameter, *χ*), individual molecular weights, and volume fractions give rise to a variety of nanoscopic structures [146,147,148].

**Figure 3:**
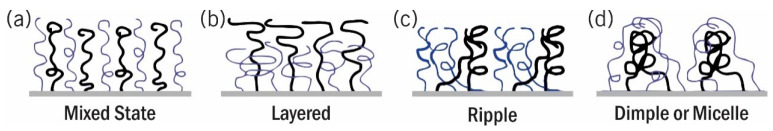
Schematics illustration of nanostructures: (**a**) mixed state, (**b**) layered, (**c**) ripple, (**d**) dimple (with periodic long-range order) or micelle (without periodic long-range order). (Reproduced with permission from Reference [55], Copyright 2009, American Chemical Society).

For MPBs, additional parameters also come into play due to the covalent fixation of one end of polymer chains on a solid surface: grafting density [149], grafting ratio [150], asymmetry of chain lengths [150], and surface geometry [55] are added into the mix and steric limitations will prefer nanoscopic over macroscopic phase segregation [149]. Phase segregation can occur vertically (perpendicular to the surface) [151], laterally (along the surface) [152], or more realistically, in a mixed state of both. Vertical phase segregation leads to a “layered” nanostructure where one component accumulates near the surface while the other component is stretched and enrichened at the outermost surface (see Figure 3b) [151]. In contrast, lateral phase segregation leads to a “ripple” structure where chemically disparate regions continuously alternate along the surface (see Figure 3c) [152]. The “dimple” phase is considered as an interplay between the “ripple” and “layered” phase where lateral and perpendicular segregation act in concert (see Figure 3d) [153]. Dimple clusters with one component at the core surrounded by the other as the shell can be periodically distributed in quadratic or hexagonal lattices on the surface. Finally, the “micelle” structure is similar to the “dimple” morphology but lacks this periodic long-range order [88].

For MPBs, polymer–surface interactions are important to consider: a silica surface may have stronger affinity to the polar component than non-polar component [69], which influences not only the required synthetic order (vide supra) but also determines the resulting morphology. For strongly different substrate interactions, a “layered” phase segregation may be observed in melt or non-selective solvents, while lateral “ripple” phases are usually favored otherwise [149].

As an additional factor, grafting density, *σ,* describes the total amount of grafted chains per unit area. The polymer chain conformation and hence thickness, *h*, of the polymer brushes is a function of this grafting density, described as *σ* = *ρhN*_A_/*M*_n_ [2]. Here, *ρ* quantifies the density of the polymer, *h* is the dry polymer brush thickness, *N*_A_ is Avogadro’s constant, and *M*_n_ is the number-averaged molecular weight. At low grafting densities, polymer chain conformations collapse (i.e., pancake or mushroom regime), while stretching away from the surface occurs as steric hindrance increases at high grafting density (i.e., stretched brush regime) [2]. The transition point between two conformations can be quantified by reduced tethered density, Σ, a dimensionless factor combining grafting density and polymer gyration radius, *R*_g_, that is described by Σ = *σπR*_g_^2^ [154]. The mushroom conformation is formed at Σ < 1, while stretched brush conformations are obtained at Σ ≫ 1. Although *σ* of MPB mainly effects the apparent brush height as that of homopolymer brushes, there is a critical MPB grafting density for phase segregation to occur [149]; akin to an order–disorder concentration in block copolymer solutions [155,156]. A mixed state is more preferred below this critical grafting density, i.e., *σ* ≪ c*/ *a*^2^*N*^1/2^, with a number of monomers in each chain, *N*; the size of a monomer, *a*; and the equilibrium concentration of monomer, c* [149].

**Figure 4:**
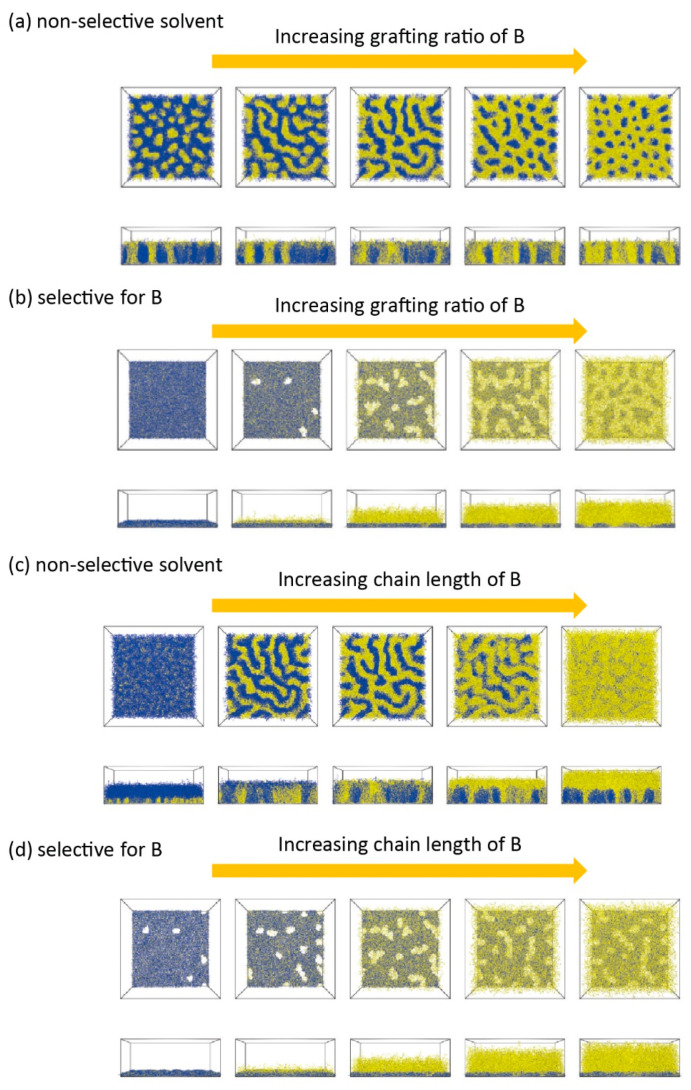
Simulation results on mixed polymer brushes (MPBs) composed of two incompatible polymers A (blue) and B (yellow). Top view and side view of MPBs with increasing grafting density of B and fixed grafting density of A in (**a**) non-selective good solvent and (**b**) selective solvent for B. The middle images represent equal grafting ratios of two components. Top view and side view of MPBs with increasing chain length of B and fixed chain length of A in (**c**) non-selective good solvent and (**d**) selective solvent for B. The middle images represent equal chain lengths of two components. (Reproduced with permission from Reference [150], Copyright 2009, American Chemical Society).

While important for phase segregation to even occur, it is the grafting *ratio*, not the grafting density, that determines which individual nanostructure results [150]. This grafting ratio describes the relative amount of grafted chains per unit area between the two components. Price et al. [99] performed a combinatorial study on the grafting ratio both computationally and experimentally (on PS/PMMA MPBs in non-selective environments). With fixed total grafting density and fixed equal degree of polymerization of both polymer brushes, only the grafting ratio was tuned. A transition along three different surface morphologies was observed in following order as grafting ratio of PS/PMMA increases from 0 to 1:1: (a) disordered state, (b) unconnected PS domains in PMMA matrix, and (c) continuous “ripple” with alternating PS and PMMA domains. In the case of selective solvent conditions, the nanostructure is mainly determined by the solvent selectivity, where the unfavored component always collapses at the substrate and the favored component accumulates at the surface regardless of grafting ratios, as demonstrated in the comprehensive study by Wang et al. [150] (Figure 4a,c). Various experimental methods can be used to control this grafting ratio (see Section 2). For example, MPBs fabricated via co-deposition approaches allow any ratio from 0% to 100% of one component, while those via Y-shaped diblock copolymers or Y-shaped surface-initiators restrict the grafting fraction by 50% (grafting ratio of 1:1). The asymmetry of MPB describes the difference of chain lengths between two components (assuming monodispersed in each components). Grafting ratio and asymmetry together describe volume fraction of two polymers in MPBs.

Finally, the geometry of the surface can have a profound effect on polymer chain conformations and, hence, the favored MPB morphology. Examples include nanoparticles (NPs) [101,157], cylindrical [158], nanorod [159,160], and nanopore [160] surfaces for which a rich variety of phase separated brush structures was predicted. Compared to planar surfaces, curvature or aspect ratio as additional parameters influence MPB phase segregation. However, such non-planar substrates are outside the scope of this contribution and the reader is referred to an excellent review by Zhao et al. [55] for more detail. In the following, we will discuss the various nanostructures under different environmental conditions and outline external factors that determine and can switch MPB morphologies.

### 3.2. Mixed Polymer Brushes Nanostructures

#### 3.2.1. Phase Segregation in Melt Condition

Seminal work on MPBs composed of two incompatible polymers in the melt was investigated using self-consistent field theory (SCFT) by Witten and Milner [151]. A “layered” nanostructure resulted from *vertical phase segregation* was achieved by separating the free chain ends to minimize the A–B interface (see Figure 3b). The “layered” structure was later found to be more favored when the chain lengths of two polymers are significantly unequal or when two polymers have strongly different interactions with the substrate [149]. Vertical phase segregation throughout the thickness of the brush often results in distinct topographies. In 1991, Marko et al. [152] discovered the *lateral phase segregation* in symmetric MPBs using SCFT. Two polymers with weak incompatibility were considered and a “ripple” topography with two gradually alternating regions was predicted. The domain size was related to and approximately twice the root-mean-square end-to-end distance, *R*_e_, of the polymer chain (where *R*_e_^2^ = 6 *R*_g_^2^) [152]. When the incompatibility of two polymer components is sufficiently strong, the phase segregation can be so significant that nearly pure A or B regions can be formed periodically along the surface [161]. 

#### 3.2.2. Phase Segregation in Non-Selective Solvents

**Figure 5:**
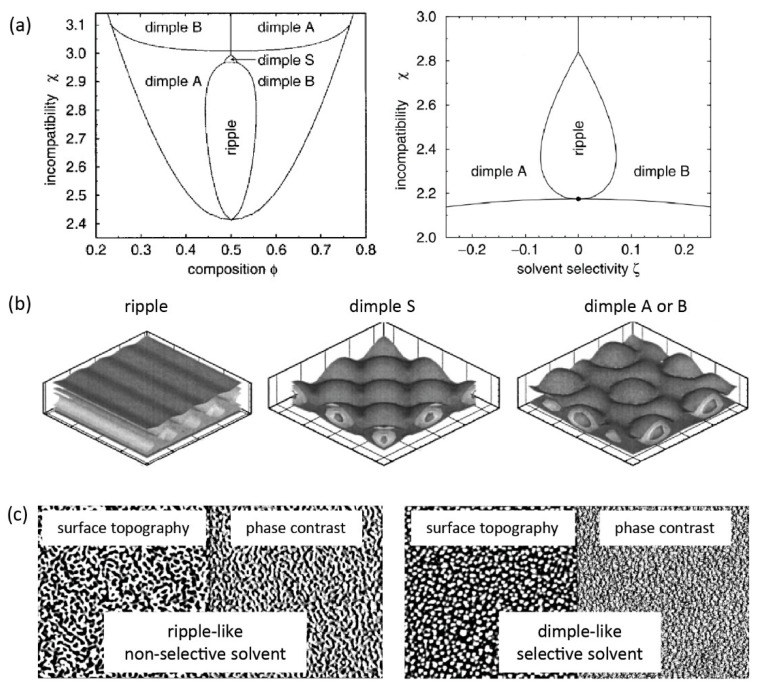
(**a**) Left: phase diagram as a function of incompatibility, *χ*, and composition, *φ*, of two polymers. Right: phase diagram as a function of incompatibility, *χ*, and solvent selectivity, ζ. (**b**) 3D illustration of “ripple”, symmetrical “dimple S”, and “dimple A or B” nanostructures from simulation results. (**c**) Atomic force microscopy (AFM) tapping mode images (5 × 5 μm^2^) of polystyrene (PS)/poly(methyl methacrylate) (PMMA) mixed brush after exposure to different solvents. (Adapted with permission from Reference [88], Copyright 2002, American Physical Society and Reference [153], Copyright 2002, American Physical Society).

In *non-selective good solvents*, “ripple” nanostructures are generally favored for symmetric MPBs, although other structure such as “layered” and “dimple” are also possible. In 1994, Lai [162] reported on MPBs in non-selective good solvents using a bond-fluctuation model. Exclusively, a periodic “ripple” structure was formed from chains clustering laterally while no “layered” structure was observed. Soga et al. [163] subsequently extended studies to various solvent conditions using a coarse-grained simulation method. The behavior of two immiscible polymers in not only good solvent but also theta- and poor solvent was studied. Lateral nanophase segregation was favored over vertical segregation for all three non-selective solvent conditions. The lateral phase segregation was reconfirmed for MPBs composed of Y-shaped diblock copolymers by Zhulina et al. [149]. Although the “ripple” structure is usually observed in symmetric MPBs, the “layered” structure is also possible in non-selective solvent but only on highly asymmetric MPBs [150,162]. If the chain length of A is longer than B, the “ripple” structure is formed near the substrate while extra chain segments of A will be forced to the outermost surface. As a result, the surface may be fully covered by the component with longer chain lengths, resembling a “layered” structure (Figure 4c) [150]. As outlined above, the “layered” structure is also possible when the two polymers have strongly different interactions with the substrate [149]. In solvents of decreasing quality yet still non-selective, the disordered and “ripple” phases are supplemented by two distinct “dimple” phases (see Figure 5a) [153]. In the “dimple *S*” phase (*S* for symmetrical), both species segregate symmetrically into clusters that arrange on a quadratically lattice (akin to a checkerboard, see Figure 5b). In the “dimple *A/B*” phase, one of the components—A or B—segregates into clusters that form a hexagonal lattice in the lateral direction, while the other component is less dense and fills the space between the clusters (see Figure 5b). The size of the lateral repeat units is approximately 1.9 *R*_e_ for the “ripple” and “dimple *S*” phases and 2.2 *R*_e_ for the hexagonal “dimple *A”* or “dimple *B”* phase. 

#### 3.2.3. Phase Segregation in Selective Solvents

**Figure 6:**
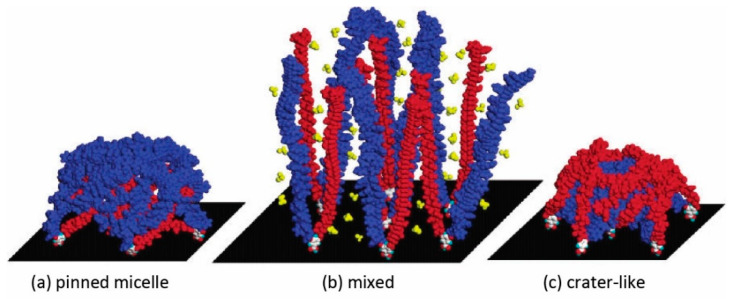
The proposed nanostructures of slightly asymmetrical Y-shaped polystyrene (PS) (blue)/poly(acrylic acid) (PAA) (red) mixed brush. (**a**) “pinned micelle” nanostructure after selective good solvent for PS (toluene) treatment, (**b**) swollen state after non-selective good solvent (mixed chloroform/methanol = 50/50) treatment, and (**c**) “crater” nanostructure after selective good solvent for PAA (water) treatment. (Reproduced with permission from Reference [76], Copyright 2003, American Chemical Society).

For MPBs composed of two incompatible polymer brushes, a selective solvent can be good for one and poor for the other component, significantly influencing surface morphologies and properties. The transition from a laterally segregated “ripple” structure to a “dimple” or “micelle” structure can be achieved by promoting perpendicular segregation through replacing a non-selective with a selective solvent [150]. According to SCFT, selective solvents favor the “dimple” morphology over “ripple” structures [88]. A phase diagram illustrates the transition between the two nanostructures as a function of incompatibility and solvent selectivity (see Figure 5a). Non-selective solvents enhance the lateral segregation and tend to stabilize the “ripple” structure, while selective solvents suppress lateral and enhance perpendicular segregation. Selective solvents enrich the preferred polymeric component at the top of the brush, whereas the unfavorable component forms “dimples”. Experiments confirm these findings: an MPB of a random copolymer of styrene and 2,3,4,5,6-pentafluorostyrene (PSF) and PMMA was prepared via sequential SI-FRP. The morphology and the chemical composition of the surface was characterized by atomic force microscopy (AFM) and X-ray photoemission electron microscopy (XPEEM), respectively. The “ripple-like” and “dimple-like” structures were observed after treatment with non-selective (toluene) and selective solvent for PMMA (acetone), respectively (see Figure 5c). However, both structures lack the long-range order which was predicted in SCFT models. The average “dimple” size obtained from AFM micrographs (2.8 *R*_e_) is also larger than that predicted by SCFT calculations (2.2 *R*_e_). They authors speculated that this difference might arise from (i) uncertainties and asymmetries in the degree of polymerization or (ii) the higher incompatibility and stretching of the experimental system compared to their theoretical calculations. “Dimple-like” structures were also observed and confirmed in other experimental studies, but still, no long-range order was observed [47,69]. This “dimple-like” structure without long-range order is sometimes also referred as “micelle.” In 2003, Julthongpiput et al. [75,76] observed “micelle” nanostructures in asymmetric binary MPBs via AFM. Asymmetric PS/PAA MPBs were prepared by anchoring slightly asymmetrical Y-shaped diblock copolymers with longer PS chains than PAA chains onto silicon substrates. As illustrated in Figure 6a, after treated by toluene, the “pinned micelle” nanostructure was obtained with PS shells covered by PAA cores. In contrast, after water treatment, the “crater” nanostructure was obtained with PAA arms on the outer walls of the “micelle” and a non-covered center area, revealing the PS core (Figure 6c). Although the “pinned micelle” structure is reminiscent of the previously predicted “dimple” phase, the “crater” nanostructure was not predicted theoretically. The authors speculated two possible reasons for the unusual “crater” morphology: the shorter PAA arms (40% PAA versus 60% PS) can facilitate clustering and the “crater” can be considered as partially covered “micelle”. Alternatively, the attractive interactions between PAA and the silicon surface may prevent conformational rearrangements of some PAA chains and keep them permanently in the immediate vicinity of the hydrophilic substrate.

The periodic “dimple” structure was predicted in computational models while the “micelle” usually appeared in corresponding experimental studies under the same conditions. The lack of periodic long-range order in experimental work was found to arise from the randomness of grafting sites. While in computational work the grafting sites of the two polymer species are usually assumed to be completely uncorrelated and uniformly distributed with a periodical pattern, while in experiments the grafting sites always appears to be randomly distributed. Wenning et al. [78] studied the effect of grafting site “randomness” using a coarse-grained bead-spring model. The density or composition fluctuations of the grafting sites was found to enhance the formation of irregular structures and randomness prevents the formation of long-range order. Even small fluctuations of the grafting sites and composition are sufficient to nucleate lateral domains within the MPBs. To examine this conclusion, Santer et al. [79] conducted a combinatorial study on effect of grafting sites randomness to the lateral segregation. Although their studies reconfirmed this conclusion using a single-chain-in-mean-field simulation, the long-range order was still not achieved experimentally. In their studies, PS/PMMA binary MPB were prepared via two methods: (i) sequential SI-FRP from co-deposited initiators and (ii) SI-ATRP followed by SI-NMP from Y-shaped bifunctional initiators. In random co-deposition, statistical fluctuations of the chemical composition occur on a local scale, while such composition fluctuations are strongly suppressed in the latter case. In contrast to predictions from simulations [78,79], no significant morphological difference was observed between the two scenarios. Hur et al. [164] later examined this discrepancy by studying the effect of minor grafting site fluctuations on the morphological structure of MPB surfaces. The ratio and position of grafting sites were varied, while the total grafting density was fixed. The resulting patterns in the microphase separated brush were observed to depend sensitively on both the wavelengths and amplitudes of the imposed grafting site fluctuations. This finding was further confirmed by Yin et al. [165] using a single-site bond fluctuation model, where the influence of the grafting site distribution on the structure of Y-shaped polymer brushes in non-selective solvents was investigated. Their calculations demonstrated that even when the MPB is composed of Y-shaped brushes, the long-range order remains limited by the distribution of grafting sites.

**Figure 7:**
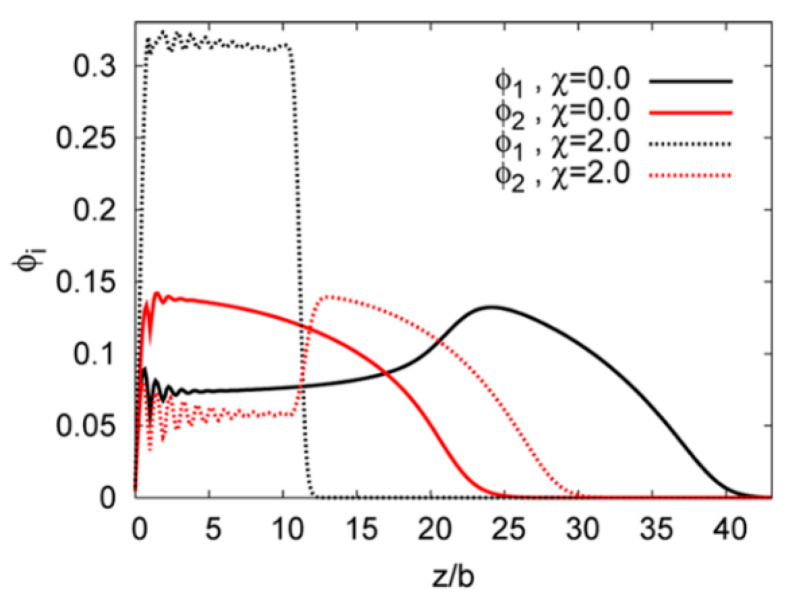
Composition profiles as obtained by 1D self-consistent field theory (SCFT) calculation on an asymmetric mixed polymer brush, with repeat units of *N*_A_ = 64 (black) and *N*_B_ = 44 (red). *χ* = 0 (solid lines) indicates non-selective solvent and *χ* = 2 (dashed lines) indicates selective solvent favoring polymer B. (Reproduced with permission from Reference [167], Copyright 2015, American Chemical Society).

“Layered” morphologies were also observed in selective solvents: in a comprehensive SCFT study by Wang et al. [150], the effects of solvent selectivity, chain length asymmetry, and grafting ratio were surveyed (Figure 4d). Density field theory (DFT) [166] and SCFT [167] further studied the vertical profiles of the two polymeric MPB components as a function of asymmetry and solvent selectivity. In contrast to non-selective solvent, where the polymer with longer chains is enriched at the top, selective solvents favor shorter chains and an inversed “layered structure” can be observed (see Figure 7). Note that profile calculations by DFT [166] and SCFT [167] were both conducted in one dimension, assuming only “layered” structures form and neglecting the details of lateral segregation.

Experimentally, both “dimple” and “layered” topologies were observed by Zhao et al. [95] via AFM on PMMA/PS binary MPBs. For a series of MPBs with a fixed PMMA and systematically varied PS molecular weight, a change in water contact angle (WCA) from 74° to 91° was observed after treatment with chloroform with the increase of PS chain length. The asymmetric system was focused where PS chain length was slightly smaller than that of PMMA. AFM studies showed that the surfaces were smooth after both chloroform (a good solvent for both PS and PMMA) and cyclohexane (a selective good solvent for PS) treatment, although different surface wettabilities were observed by tensiometry. In contrast, rough surfaces resulted from relatively ordered nanoscale domains after treatment with glacial acetic acid (a selective good solvent for PMMA). XPS studies confirmed that the PMMA chains were enriched at the outermost layer. The changes in surface topology and wettability were found to be reversible as a function of solvent treatment. A similar behavior was found for thermal annealing, where the same surface topology and wettability were found after cyclohexane treatment [96]. Based on observations above, the authors proposed a “layered” structure for cyclohexane-treated surface and “micelle” structure for glacial acetic acid-treated surface. Although both glacial acetic acid and cyclohexane are selective solvents, different surface morphologies were observed. The authors speculate this difference may result from the differences in polymer–solvent interactions (i.e., glacial acetic acid is a much better solvent for PMMA than cyclohexane for PS).

## 4. Phase Behavior of Ternary Mixed Brushes

**Figure 8:**
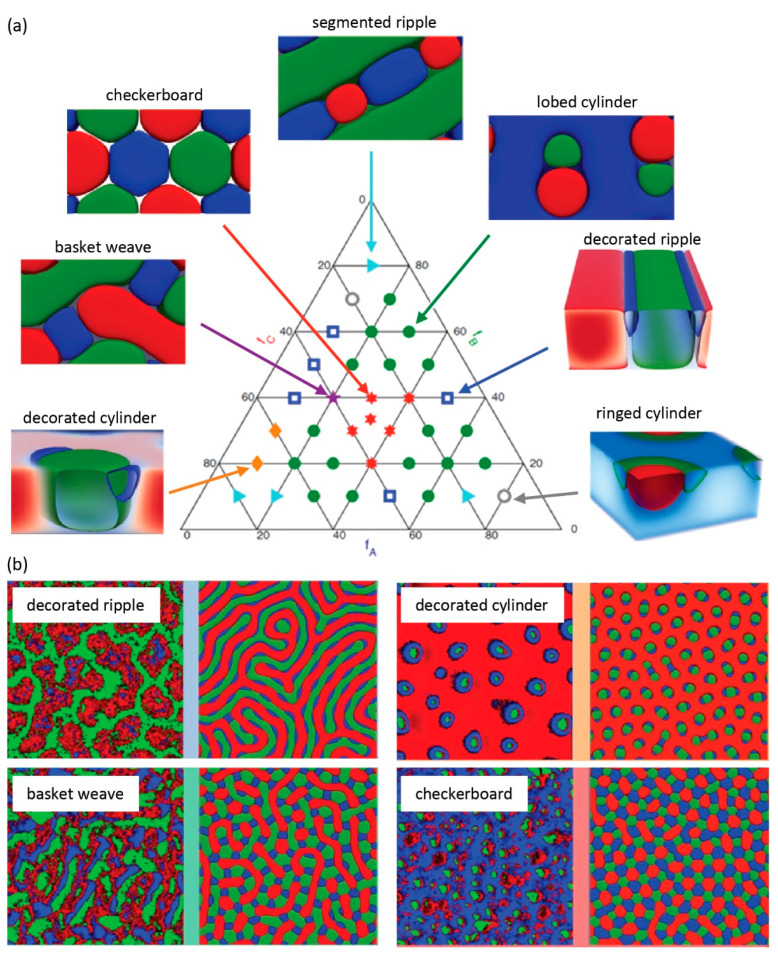
Nanostructures of ternary mixed polymer brushes: (**a**) phase diagram of possible nanostructures as a function of volume fraction of three components, (**b**) a comparison between (left) atomic force microscopy (AFM) result on polystyrene (PS)/ poly(methyl methacrylate) (PMMA) / poly(4-vinyl pyridine) (P4VP) and (right) simulation result of the corresponding volume fraction. (Reproduced with permission from Reference [43], Copyright 2016, American Chemical Society).

Recent computational studies have added complexity and elaborated on the phase segregation behavior of *ternary* brushes composed of *three* different polymeric species [43]. As to be expected, a much richer phase behavior can be observed: SCFT studies predict a total of *seven* nanostructures (see Figure 8a). Of these seven, four were observed experimentally in PS/PMMA/P4VP mixed brushes that were synthesized via sequential SI-FRP and characterized by AFM (See Figure 8b).

Although a good agreement was found between computational and experimental studies, the limitations of sequential SI-FRP remain. The authors modified the volume fractions of individual components by tuning the reaction times of each step. As we discussed above, the volume fraction is a complex factor combining grafting ratio and asymmetry of chain lengths. The slow initiation (half-life of ACVA ≈ 10h at 65 °C [43]) and fast propagation of SI-FRP is anticipated to determine the grafting ratio between the three components. However, the free radical polymerization also results in highly asymmetric brushes (e.g., 98.0, 93.6, 181.3 kg/mol for PS, PMMA, P4VP as reported by GPC, respectively). This later was found to significantly affect the resulting structure [44]. Finally, the SCFT model was based on monodisperse polymer chains, which is far away from the capability of SI-FRPs. Nonetheless, this seminal study on ternary MPBs provides intriguing new pathways towards hitherto undiscovered morphologies and dynamic surfaces.

## 5. External Stimuli

The reversible switching of MPB surfaces has been widely observed as a function of various stimuli. As we will describe below, switching follows two major mechanisms: (a) *both* components respond to external stimuli selectively with phase segregation resulting from their different affinity to the environment or (b) *one* of the two component responds to external stimuli, while the other component facilitates the switching or provides additional physical or chemical properties. Most switching experiments have been triggered by selective organic solvents. Recent studies showed that such surface switching property can also be achieved by “greener” external stimuli, including but not limited to, temperature, pH, or ion strength in aqueous solutions. The following compiles various such external influences that have reportedly altered MPB surface properties.

### 5.1. Solvents

A reversible switching of surface properties on PS/P2VP mixed brushes upon exposure to different solvents was observed by Sidorenko et al. [85]. Toluene and acidic water (pH < 4) were used as selective solvents for PS and P2VP, respectively, with surface-enrichment of the individual components characterized via XPS and tensiometry. The hydrophobic surface (water contact angle, WCA = 81.3°) after toluene treatment indicated a PS-rich surface while a hydrophilic surface (WCA = 13.8°) after acidic water treatment indicated a P2VP-rich surface. An alkaline bath (0.1 N NH_4_OH) and THF were used to return the layer properties back to the original state (WCA = 71.5°). The reversibility of this switching was confirmed by repeating this cycle three times. A fine tuning of surface hydrophilicity (i.e., anywhere from WCA = 63.3° to 88.1°) was found to be possible by treating PS/P2VP mixed brushes with ethanol/toluene mixtures of different concentrations [87]. In another example, an MPB composed of poly(methyl acrylate) (PMA) and PSF exhibited a reversible switching of surface mechanical properties upon exposure to different solvents [93]. PSF and PMA are mechanically dissimilar, i.e., glassy and rubbery, respectively, at room temperature. The reversible switching between glassy and rubbery surfaces with different elastic moduli was achieved by treating with toluene (selective for PSF) or acetone (selective for PMA), respectively. Naturally, this selective solvent approach encompasses a wide scope of polymer combinations, e.g., PS/PMMA, PAA/PS, and PS/PPhOXA as summarized in Table 1. It is important to note that, although the surface switching occurs in a selective solvent, the MPB is always utilized or characterized in a different environment. MPB films should be dried rapidly following solvent treatment so that the resulting film morphology can be “frozen” in. At ambient conditions, if the polymers in the dry film are in a glassy state, then the film morphology can be stable for a long period of time [69]. A locking/unlocking mechanism was also further studied for MPB exposing to selective solvent [168,169]. Two types of MPB were predicted by DFT calculation as a function of chain length and incompatibility. A W-type brush can memorize the solvent-induced surface enrichment of one component, even upon treatment with a solvent favoring the other component which can be seen as a kind of lock state. The switching ability to another surface enrichment has to be unlocked by non-selective good solvent as a key. In contrast, a U-type brush is lockless and able to adapt to the environment without the necessity of non-selective solvent treatment.

### 5.2. pH and Ion Strength

The pH and ion strength in aqueous solutions were also used to switch surface composition and properties. Aqueous solutions provide a more environmentally friendly approach when compared to organic solvents. A mixed polyelectrolyte brush composed of two oppositely charged polymers, PAA and P2VP, was observed to be responsive to pH [70]. At small pH, P2VP chains are protonated and stretch away from the surface due to the electrostatic repulsion along their backbone. In contrast, at large pH, negatively charged PAA chains extend and decorate the surface for similar reasons. Surface enrichment was evidenced using tensiometry and zeta potential experiments as a function of pH. Recently, ion strength was also used to switch PAA/ PEO MPB surfaces for protein selective adsorption [50]. PAA brush swelling is driven electrostatic interactions and osmotic pressure. In the osmotic regime, counterion condensation inside the brush leads to a stretched chain conformation and surface enrichment with PPA. As the ion strength further increases, PAA chains enter the salted regime and collapse at the bottom, while PEO chains are exposed on top. The reversible adsorption and desorption of proteins was regulated by switching ion strength between 10^-3^ M and 0.15 M, respectively. 

### 5.3. Dry/Wet Condition

Motornov et al. [72] prepared a switchable MPB composed of two low *T*_g_ polymers, poly(dimethylsiloxane) (PDMS) and a highly branched ethoxylated poly(ethylenimine) (EPEI). PDMS/EPEI MPB film was observed to switch rapidly and reversibly between hydrophilic and hydrophobic states upon contact with water and air, respectively, and PDMS/EPEI MPBs are responsive without the addition of any acid, base or salt. Under water, hydrophilic EPEI clusters swell and the polymer protrudes to the polymer brush/water interface to form a hydrophilic surface. As soon as the MPB surface is removed from water, the film loses some fraction of water while remaining swollen. The brush surface is immediately covered by the hydrophobic PDMS shell with swollen EPEI core underneath and switches from hydrophilic to hydrophobic state. In air, the film loses water slowly until an equilibrium state is reached that is determined by the ambient air’s relative humidity. At the same time, PDMS shells form a hydrophobic surface with a water contact angle higher than 90° which protects the film from wetting in humid air. As a result, the film is non-wettable in humid air (up to 95% relative humidity) and retains its hydrophobic properties. The film transitions into the hydrophilic state only if in contact with water. 

### 5.4. Temperature

Chemically non-invasive external stimuli (e.g., temperature, magnetic, electric fields) prevent the waste of solvents and allows facile in situ reversible switching. They can also provide promising alternatives for biological applications, where invasive stimuli can be detrimental to the living organism. Estillore et al. [111] reported a temperature/solvent dual responsive poly(*N*-isopropylacrylamide) (PNIPAm)/PS mixed brush on a free-standing polymer film. PNIPAm is a well-known temperature-responsive material with a lower critical solution temperature (LCST) of 32 °C. Above LCST, PNIPAm brushes are collapsed in water while swollen below this temperature. However, this initial work was mainly focused on the responsiveness to solvent polarity and only few temperature-responsive behaviors were discussed. As the temperature was elevated above LCST of PNIPAm, the free-standing film turned increasingly opaque while remaining swollen in water. This opaqueness was attributed to the conformational transition of the PNIPAm brushes from coil to globule. A more comprehensive study on PNIPAm-based mixed brushes was conducted by simulation [68]. A system was designed to mimic PEO/PNIPAm mixed brushes using a coarse-grained model. PEO is inherently temperature-responsive with a swelling behavior that is antagonistic to that of PNIPAm. The increase in the fraction of PEO chains strongly modifies swelling of the brush in the temperature range below and around LCST. For T < LCST, water behaves as selective solvent for PNIPAm and PNIPAm dominates the MPB surface, shielding PEO chains from the aqueous environment. For T > LCST, water becomes a selective solvent for PEO and an inversed vertical segregation is observed. 

## 6. Applications

MPBs provide two major advantages: (a) allowing two immiscible polymers to coat a single surface without macro-phase separation and/or de-wetting, and (b) providing a reversible switching ability between two or more physical properties. Additionally, versatile synthetic routes allow fabrication of MPB on substrates with varied geometry, including planar surfaces [48], nanoparticles [170], and even porous materials [45,46]. As such, numerous applications for MPB films across countless fields have been suggested and described, including but not limited to nanomotors [47], surface friction modifiers [91], nanovalves [48], drug release [45], catalysis [46], and controlled adsorption/desorption of polymers [49] or proteins [50,51,52,53,54].

In organic electronics, A PDMS/P2VP mixed brush on a planar indium tin oxide (ITO) electrode was used as nanovalves for electrochemical gating [48]. MPB morphological transitions in response to changes in pH resulted in the opening, closing, or precise tuning of permeability for ion transport through the channels that are formed in the nanostructured thin film layer. P2VP homopolymer brushes were swollen and allowed permeation for anions in the protonated state at pH 2. In contrast, at pH 6, P2VP chains collapsed near the substrate and blocked the diffusion of anions. Nanovalves formed by the mixed brush were found to regulate ion transport more efficiently than homopolymer brush films. In P2VP homopolymer brushes, “leaking points” can be formed upon polymer chain collapse in a poor solvent. Introducing the PDMS component helped seal these possible “leaking points” and improved nanovalve permeability control.

In a “smart” drug delivery system, PHPMA/PDEAEM mixed brushes on mesoporous silica nanoparticles (MSNs) were designed as a gatekeeper to control the loading and release of guest molecules to target pathological cells [45]. The drug loading/release ability is mainly attributed to the pH-response of PDEAEMA, which was used to demonstrated controlled release of doxorubicin (Dox) as model drug. In acidic solution, the positively charged PDEAEMA becomes hydrophilic and opens the MSN core, while in basic solution neutralized PDEAEMA becomes hydrophobic and aggregates on the surface to close the pores. The critical pH for PDEAEMA for this switching was determined as pH 6.5, which is lower than that of human blood (pH 7.4). This assures that the release of any MSN-polymer encapsulated drug can only be triggered after reaching the pathological cells where the pH is slightly more acidic. Biocompatibility of PDEAEMA homopolymer brushes was also increased through combination with PHPMA as MPB to enhance the hydrophilicity and biocompatibility of MSN.

In catalysis, poly(styrene sulfonic acid) (PSSA)/poly(vinyl imidazolium chloride) ionic liquid (PIL) mixed brushes on ceramic membrane substrates were used for biomass hydrolysis [46]. Hydrolysis is mainly catalyzed by PSSA chains while neighboring PIL chains help solubilize lignocellulosic biomass and thereby enhance the catalytic activity of the PSSA. Cellulose hydrolysis in ionic liquid was confirmed by high total reduced sugar (TRS) yields, suggesting MPB can potentially serve as an alternative catalyst for the more expensive and slower cellulase enzymes. As an additional benefit, the catalytic activity was tunable by varying the polymer brush chain length and grafting density. In another example, stimuli-responsive catalytic activity has been achieved by PNIPAm/P2VP mixed brushes on palladium and platinum nanoparticles [170]. The catalytic activity of the nanoparticles was tested by a model reaction, reduction of 4-nitrophenol with NaBH_4_. The P2VP homopolymer brushes on metallic particles was evidenced to be catalytic for the reduction reaction. The temperature-responsive behavior of PNIPAm introduced the responsive capability to the nanoparticle catalyst. With stretched PNIPAm chains below LCST, the mixed brushes exhibited a similar catalytic behavior as P2VP homopolymer brushes. Upon heating above LCST, the collapse of PNIPAm creates a diffusional barrier for the reactants, resulting in a decreased reaction rate.

For biological applications, MPBs can selectively adsorb and release proteins. Theoretical studies have demonstrated protein adsorption or resistance behavior on homopolymer (e.g., PEO) brushes [171,172,173]. The dynamic character of MPBs allowed for switchable protein adsorption and desorption that can be triggered by external stimuli [50,51,52,53,54]. For example, a reversible adsorption/desorption switching behavior can be achieved by combining the protein-repellent properties of PEO and the stimuli-responsive adsorption behavior of PAA on planar gold substrates [50]. Modification of ion strength- and pH-controlled protein adsorption is mainly governed by the swelling/collapse behavior of anionic PAA chains through electrostatic interactions. Further, the behavior of three proteins, human serum albumin (HSA), lysozyme (Lys), and human fibrinogen (Fb) with very different sizes, shapes, and isoelectric points was investigated on PEO/PAA mixed brushes. HSA adsorption is negligible on the pure PAA and on the mixed PEO/PAA brushes. Swollen PAA chains selectively adsorb Lys and Fb, while collapsed PAA chains will expose PEO chains and repel any proteins. These adsorption/desorption processes can be cycled changing ion strength, and the extent of adsorption/desorption can be finely tuned by adjusting the PAA/PEO composition. Selective adsorption and complete desorption make such systems potentially reusable detectors or separators for certain proteins. 

## 7. Challenges and Future Directions

Although development of synthetic routes featuring SI-FRPs, SI-RDRPs and SI-ROPs has provided versatile chemistry platforms, there remains room for improvement. The “living” chain ends in sequential SI-RDRPs may interfere with each other and result in unexpected side reactions. While these synthetic limitations have been addressed by combining SI-RDRP and SI-ROP, orthogonal polymerizations are limited in monomer scope to one vinyl and one cyclic monomer.

While the groundwork has been laid by a body of impressive work from previous researchers, there remains room for improvement to help pave the road for MPBs to transition into industrial application and scale-up. For example, recent developments in polymer brushes synthesis, including oxygen tolerance [60,81], metal-free polymerization [82], and external regulation [81,83,84,174,175,176,177,178,179,180] have the potential to make MPB fabrication more user friendly than ever before. Newly described orthogonal approaches in solution will increase synthetic precision: in contrast to sequential SI-FRP/SI-RDRP, orthogonal polymerization techniques and “click” reactions do not compete for the same initiating sites and improve control over grafting ratios. 

Another remaining challenge is the characterization of the individual polymer lengths in sequential SI-P. As the asymmetry of chain lengths effects the resulting nanostructures, it is important to be able to target and characterize the degrees of polymerization of *both* polymers to obtain precise topographical control. Some studies estimate polymer brush chain lengths by using GPC to characterize the molecular weight free polymers from sacrificial initiators in the reaction mixture to then compare them to the grown brush heights [95]. However, this approach is controversial: Genzer and co-workers [181] pointed out that the free polymers in solution grow faster than polymers initiated from the surface, in part due to decreased steric hindrance during monomer addition. Further, the rate of surface-initiated polymerization depends on the density of grafting sites. Considering the steric limitations induced by the first polymerization, the reaction rate of a second polymerization may differ even further from that of the free polymerization in solution. Ellipsometry has also been attempted to quantify the growth of second polymer by characterizing film thickness change after second polymerization [181]. However, film thickness of polymer brushes is a function of many factors, such as grafting density, and inherently neglects dispersity. A doubled film thickness after second polymerization does not necessarily indicate equal chain lengths of two polymers. 

Further, direct experimental evidence for the 3D nanostructures remains prohibitively challenging. Various nanostructures have been proposed computationally and experimentally, each of which represents a mixed state of both lateral and vertical phase segregation. While characterizing their outermost surface is feasible via AFM [43,93], this technique is often limited to specific MPB systems composed of two mechanically distinct polymers (e.g., PMA/PSF [93]). It remains difficult to characterize chemical compositions disregarding differences in elastic moduli. To overcome this limitation, scattering scanning near-field infrared microscopy (s-SNIM) was successfully used in isolated studies to observe the lateral segregation of PS/PMMA mixed brushes [98]. The s-SNIM combines AFM with infrared laser spectroscopy, simultaneously providing both topographic and chemical information and was able to differentiate between PS-rich and PMMA-rich areas on the surface. Recently, cryo-electron microscopy (cyroEM) and cryo-electron tomography (cryoET) were used to directly observe nanostructure of two components in MPB on nanoparticles.

In contrast to topography, determining the polymer brushes’ vertical distribution is more challenging. Traditional hard X-ray scattering techniques rely on electron density contrast, which can be very low for MPBs as they are comprised of two (or more) carbonaceous low *electron density* materials. Here, the selective enhanced contrast that resonant soft X-ray scattering (RSoXS) provides can be leveraged to develop a comprehensive picture of phase segregation within the MPB. The feasibility of this concept was highlighted through previous studies on *spin-coated* PS/PMMA bilayers [182] and PS-*b*-PMMA diblock copolymer films [183] that show distinct features under in RSoXS and support the notion that the technique will be a powerful tool for advanced MPB characterization.

## 8. Conclusion and Outlook

This review outlines research on MPBs as responsive materials to provide tremendous potential for engineering “smart” substrates and interfaces. The MPBs have been used to modify varied materials such as silicon, silicon oxide, gold, metal oxide, carbon nanotube, glass, ceramic, and polymer. Further, substrates with different geometries, including planar surface, nanoparticle, nanotube, membrane, and free-standing films have been modified. This versatility illustrates the vast opportunities that their ability for surface-switching upon exposure to external stimuli provides to impact on both established and numerous future applications. We therefore hope that our excursion into previous work and the current limitations of MPB surfaces can inspire future directions in this fruitful field. In conclusion, we encourage studies on the precise characterization and more facile synthetic fabrication which is essential for reproducibility and quality control in industrial manufacturing—a gap that will hopefully be bridged soon to ultimately deliver this dynamic surface concept from the academic lab to industry and into our daily lives.

## Figures and Tables

**Table 1:** An overview over mixed polymer brushes studies.

Polymer Combination	Synthetic Approach	Surface Initiator	Substrate	Stimuli	Reference
PEO/PAA	“grafting to”	N/A	gold	Ion strength	[50]
PDMS/EPEI	“grafting to”	N/A	Si	dry/wet	[72]
PDMS/P2VP	“grafting to”	N/A	Si/ITO	pH	[48]
PAA/P2VP	“grafting to”	N/A	Si	pH	[70]
PS/P2VP	sequential SI-FRP	non-selective initiator	Si, polyamides film	solvent	[85,87,88,89]
	“grafting to”	N/A	Si, silica NP		[69,90,91,92]
PSF/PMMA	sequential SI-FRP	non-selective	Si	solvent	[88,93]
PMMA/PS	sequential SI-ATRP/SI-NMP	co-deposited	Si, silica	solvent	[79,94]
		Y-shaped			[79,95,96]
	sequential SI-FRP	non-selective			[47,97,98,99]
PS/PMMA/P4VP	sequential SI-FRP	non-selective	Si	N/A	[43]
PAA/PS	sequential SI-FRP	non-selective	Si	solvent	[88]
	“grafting to” (Y-shaped diblock)	N/A			[75,76]
	sequential SI-ATRP/SI-NMP	Y-shaped	silica NP		[100,101]
PS/P4VP	sequential SI-ATRP/SI-NMP	co-deposited	silica	solvent	[102]
		Y-shaped			
PtBA/PS	sequential SI-ATRP/SI-NMP	Y-shaped	silica NP	N/A	[103,104,105,106,107,108,109]
PHPMA/PDEAEMA	sequential SI-ATRP/SI-RAFT	Y-shaped	MSN	pH	[45]
PHEAA/PMETA	sequential SI-ATRP/SI-PIMP	co-deposited	Si	N/A	[110]
PSSA/PIL	sequential SI-ATRP/SI-FRP	co-deposited	glass, ceramic membrane	N/A	[46]
PNIPAm/PS	sequential SI-ATRP/SI-FRP	Layer-by-Layer deposited	Si, free standing polymer film	solvent, temperature	[111]
PS/PεCL	one-pot SI-ATRP/SI-ROP	co-deposited	CNT	solvent	[112]
	sequential SI-ROP/SI-NMP	Y-shaped	silica NP		[113]
PMMA/PLLA	one-pot SI-ATRP/SI-ROP	co-deposited	CNT	solvent	[112]
PS/PPhOXA	one-pot SI-NMP/SI-ROP	Y-shaped	Si	solvent	[114]

PEO = poly(ethylene oxide), PAA = poly(acrylic acid), PDMS = poly(dimethylsiloxane), EPEI = ethoxylated poly(ethylenimine), P2VP = poly(2-vinyl pyridine), P4VP = poly(4-vinyl pyridine), PSF = random copolymer of styrene and 2,3,4,5,6-pentafluorostyrene, PMMA = poly(methyl methacrylate), PS = polystyrene, PtBA = poly(tert-butyl acrylate), PHPMA = poly(N-(2-hydroxypropyl)methacryl-amide), PDEAEMA = poly(2-diethylaminoethyl methacrylate), PHEAA = poly(*N*-hydroxyethyl acrylamide), PMETA = poly(2-(methacryloyloxy) ethyl trimethylammonium chloride), PSSA = poly(styrene sulfonic acid), PIL = poly(vinyl imidazolium chloride) ionic liquid, PNIPAm = poly(*N*-isopropylacrylamide), PεCL = poly(ε-caprolactone), PLLA = poly(L-lactic acid), PPhOXA = poly(2-phenyl-2-oxazoline), ITO = indium tin oxide, NP = nanoparticle, MSN = meso-porous silica nanoparticle, CNT = carbon nanotube.
